# Genetic Modifiers of Non-Penetrance and RNA Expression Levels in *PRPF31*-Associated Retinitis Pigmentosa in a Danish Cohort

**DOI:** 10.3390/genes14020435

**Published:** 2023-02-08

**Authors:** Kristian Lisbjerg, Karen Grønskov, Mette Bertelsen, Lisbeth Birk Møller, Line Kessel

**Affiliations:** 1 Department of Ophthalmology, Copenhagen University Hospital-Rigshospitalet, 2600 Glostrup, Denmark; 2 Department of Clinical Genetics, Copenhagen University Hospital-Rigshospitalet, 2100 Copenhagen, Denmark; 3 Department of Clinical Medicine, University of Copenhagen, 2200 Copenhagen, Denmark

**Keywords:** retinitis pigmentosa, RP11, *PRPF31*, non-penetrance, MSR1, *CNOT3*, gene expression

## Abstract

(1) Background/aims: To examine potential genetic modifiers of disease penetrance in *PRPF31*-associated retinitis pigmentosa 11 (RP11). (2) Methods: Blood samples from individuals (n = 37) with *PRPF31* variants believed to be disease-causing were used for molecular genetic testing and, in some cases (n = 23), also for mRNA expression analyses. Medical charts were used to establish if individuals were symptomatic (RP) or asymptomatic non-penetrant carriers (NPC). RNA expression levels of *PRPF31* and *CNOT3* were measured on peripheral whole blood using quantitative real-time PCR normalized to *GAPDH*. Copy number variation of minisatellite repeat element 1 (MSR1) was performed with DNA fragment analysis. (3) Results: mRNA expression analyses on 22 individuals (17 with RP and 5 non-penetrant carriers) revealed no statistically significant differences in *PRPF31* or *CNOT3* mRNA expression levels between individuals with RP and non-penetrant carriers. Among 37 individuals, we found that all three carriers of a 4-copy MSR1 sequence on their wild-type (WT) allele were non-penetrant carriers. However, copy number variation of MSR1 is not the sole determinant factor of non-penetrance, as not all non-penetrant carriers carried a 4-copy WT allele. A 4-copy MSR1 mutant allele was not associated with non-penetrance. (4) Conclusions: In this Danish cohort, a 4-copy MSR1 WT allele was associated with non-penetrance of retinitis pigmentosa caused by *PRPF31* variants. The level of *PRPF31* mRNA expression in peripheral whole blood was not a useful indicator of disease status.

## 1. Introduction and Background

*PRPF31*-associated retinal dystrophy, or retinitis pigmentosa 11 (RP11), is an autosomal dominantly inherited type of retinitis pigmentosa [1]. Retinitis pigmentosa (RP) is a genetically heterogeneous disorder, and variants in multiple different genes can be involved as the underlying cause of the disease [2]. Moreover, because of differences in genetic etiologies, disease mechanisms and clinical presentation in RP may differ [3]. RP is a progressive disorder, and the main symptoms are night blindness, constriction of visual fields, loss of visual acuity, and eventually, it can cause blindness. RP is a relatively rare condition, with a worldwide prevalence of around 1 in 4000 [4,5]. *PRPF31* variants account for 5–10% of all dominant RP cases [6]. The primary pathogenic mechanism and cause of symptoms in RP11 cases are thought to be haploinsufficiency, where one wild-type allele is insufficient to drive normal protein production [7,8]. RP11 is characterized by a variable disease onset and an exponential decline of visual fields of 8–10% per year leading to legal blindness, and an incomplete disease penetrance is reported despite the dominant inheritance pattern [9,10,11].

Novel therapeutic options for inherited retinal diseases (IRDs) are advancing, as illustrated by the approved gene augmentation therapy voretigene-neparvovec for RPE65-related retinal dystrophy [12]. Additionally, they provide a dramatic change in the future perspectives for individuals with IRDs. A scientific emphasis on disease mechanisms is key for developing the best strategies for future treatments in the genetically diverse spectrum of RP and IRDs in general [13].

The *PRPF31* gene encodes a pre-mRNA splicing factor involved in the coupling of the spliceosome. The gene spans 16 kb on chromosome 19q13.4 and consists of 14 exons encoding a protein of 499 amino acids [14]. The spliceosome modulates pre-mRNA splicing where the non-coding introns are removed and the exons are joined together to form mature mRNA [15]. Splicing of pre-mRNA is a critical step in protein synthesis, and *PRPF31* is ubiquitously expressed in all tissues [16]; however, disease-causing variants in *PRPF31* only result in retina-specific and non-syndromic disease [17,18]. Genetic variants in other genes encoding for splicing factors (*PRPF3*, *PRPF4*, *PRPF6*, *PRPF8*, *RP9*, *snRNP200*) can also lead to autosomal dominant RP, but *PRPF31* variants are the most frequent cause of splicing-factor-related RP [19]. The majority of previously reported disease-causing variants in *PRPF31* are truncating variants, resulting in nonsense mediated decay (NMD), or are null alleles [20,21], and this corresponds well with a disease mechanism of haploinsufficiency.

Non-penetrance has been observed in several families, leading to asymptomatic carriers of pathogenic *PRPF31* variants [20]. Increased *PRPF31* expression from the wild-type (WT) allele is hypothesized to be the explanatory factor for the non-penetrant carriers in RP11 families [22], and the importance of the WT allele was first observed in 1997 [23]. In this context, one might envision a theoretical threshold value for *PRPF31*, over which RP would be prevented, and below which the disease would manifest as RP [20]. Various genetic factors, both cis and trans, are thought to influence *PRPF31* mRNA expression levels. One factor is a minisatellite repeat element (MSR1) located adjacent to the *PRPF31* promoter [24]. The MSR1 is found as normal variants with three or four copies (copy number variation, CNV); a 4-copy WT allele is thought to be related to non-penetrance [24]. Another modifier gene, *CNOT3,* is also located at chromosome 19q13.4, 20 kB downstream to *PRPF31*, and is part of the Ccr4-Not complex involved in gene expression regulation. A high *CNOT3* gene expression has been suggested to suppress *PRPF31* expression [25], thus a relatively low level of *CNOT3* may be associated with non-penetrance, but reports on this topic are contradictory, as others have not found this association [26].

In this study, we aimed to examine *PRPF31* expression levels and possible modifiers in symptomatic and non-penetrant carriers of disease-causing variants in *PRPF31* in a Danish cohort. With only one wild-type allele, the expression level of *PRPF31* is predicted to be reduced, but it is unknown if disease presence or severity can be predicted from mRNA expression levels in peripheral whole blood. With mRNA expression analyses, we aimed to explore and investigate if a simple blood test can be used to identify a threshold for symptomatic disease. Furthermore, we aimed to investigate if MSR1 copy number variation on the WT allele is a useful predictor of disease penetrance, which could benefit the genetic counseling of RP11 families.

## 2. Materials and Methods

### 2.1. Subjects

**Figure 1:**
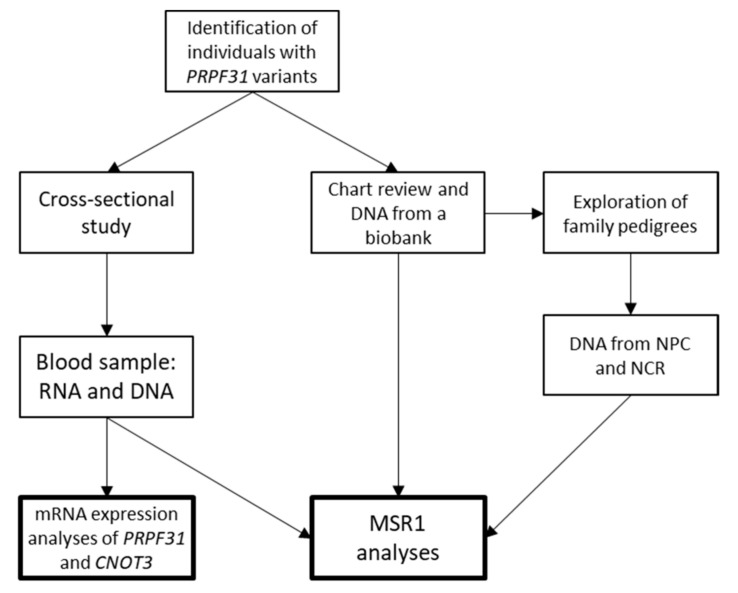
Illustration of the study workflow. mRNA expression analyses were dependent on whether the individual had a blood sample collected in a PAX tube. NPC: non-penetrant carrier. NCR: non-carrier relative.

Patients with RP caused by variants believed to be disease causing in the *PRPF31* gene and their non-symptomatic first-degree relatives were included in the study. Participants were identified from national registries and previous studies [9,27]. Clinical information on RP status was confirmed from medical files. Relatives carrying the *PRPF31* variant without any subjective symptoms of RP were identified as non-penetrant carriers (NPC). As not all NPCs had available clinical information, we chose to define NPC in this study from a patient-centered point-of-view and used the individuals’ subjective complaints as the starting point for inclusion as an NPC. The asymptomatic individuals experienced no night blindness, had no visual field issues, or other visual complaints. Relatives not carrying the family-specific *PRPF31* variant were identified as non-carrier relatives (NCR). Some patients and relatives delivered a fresh blood sample, whereas others contributed with DNA samples obtained previously in relation to the clinical work-up of the family, see Figure 1. DNA samples had been stored at the biobank for hereditary eye diseases at the Kennedy Center. mRNA expression analyses were only possible if the subjects had participated in the clinical cross-sectional study, and thereby had their blood sample collected in the proper container for RNA isolation (PAX tube). The study was conducted at the Department of Ophthalmology and the Department of Clinical Genetics at Copenhagen University Hospital, Kennedy Center.

### 2.2. Genetic Testing and Classification

The majority of the molecular genetic testing had been performed in a previous study [27] using a next generation sequencing (NGS) panel of 125 genes known to be related to retinal dystrophies. For some individuals the genetic testing had been performed as part of clinical routine diagnostics, with a retinal dystrophy panel of 186 retinal dystrophy genes. A few samples had been analyzed by external laboratories. All variants were reevaluated and classified using the American College of Medical Genetics and Genomics (ACMG) classification [28], as well as updated ClinGen sequence variant interpretation working group recommendations (https://www.clinicalgenome.org/working-groups/sequence-variant-interpretation/, accessed on 29 December 2022). MLPA analysis was performed using the MRC-Holland kit P235 when a deletion or duplication was suspected from CNV analysis using NGS data (MRC-Holland, Amsterdam, Netherlands). Sanger sequencing or MPLA analysis, targeting the family-specific variant, was used to confirm variant status in relatives.

### 2.3. RNA Expression Analysis of PRPF31 and CNOT3

The expression levels of *CNOT3* and *PRPF31* were measured in attending participants (RP, n = 17 and NPC, n = 5) using quantitative real-time PCR (qRT-PCR). RNA was isolated from whole blood (in PAX tubes) using CMG-1084 total RNA kit including DNase treatment and analyzed on Chemagic 360 instrument (PerkinElmer, Waltham, MA, USA). Following RNA isolation, the RNA samples were kept at −80 degrees Celsius until use. Random hexamer primers were used for cDNA synthesis. Subsequently, amplification of cDNA, using FAM-labeled TaqMan probes and primers, specific for *CNOT3* (Hs00248115_m1, Applied Biosystems, Waltham, MA, USA) and *PRPF31* (Hs00210306_m1, Applied Biosystems, Waltham, MA, USA), was performed for the relative quantification of mRNA for the two genes. For normalization, a FAM-labeled TaqMan probe and primers specific for the endogenous *GAPDH* (Hs99999905_m1, Applied Biosystems) were used. The relative standard curve method was used to perform a relative quantification of the gene expression. Standard curves for *PRPF31*, *CNOT3*, and *GAPDH* assays were prepared using five samples of control cDNA, performed by threefold serial dilutions. All measurements were performed as technical triplicates. In this study we defined the expression level in the RP group as the reference when calculating fold-change to the non-penetrant carriers’ expression levels.

### 2.4. Copy Number of MSR1

For the MSR1-analysis, we analyzed DNA from all available family members to allow for analysis of segregation with disease or WT allele, see Supplementary Figure S1. A standard PCR reaction, with the primer 5′ FAM-GTTAGGGGTTTGGACTGC 3’ and a reverse primer 5’ GATGTGGCCACCAAATAC 3’, was used to perform PCR fragment analysis (AmpliTaq Gold, Applied Biosystems, Waltham, MA, USA), the number of copies of the MSR1 repeat element was determined by capillary electrophorese on ABI3730 equipment (Illumina, San Diego, CA, USA). The GeneMapper software (Applied Biosystems, Waltham, MA, USA) was used for the analysis. The 3-copy allele peak was at 462 bp, 4-copy allele peak at 500 bp. The method was previously reported by Rose et al. [24].

### 2.5. Statistical Methods

All analyses were performed with the statistical software R (R core team, Vienna, Austria) [29]. The relative gene expression results are reported as mean ± standard deviation (SD). Student’s *t*-test was used to compare the normalized relative quantified expression levels in the RP group with those in the non-penetrant carrier group. A *t*-test can be applied in small sample sizes [30] and the calculations were repeated with the non-parametric Wilcoxon’s rank sum test confirming similar results. The correlation between mRNA expression levels of *PRPF31* and *CNOT3* was described with Pearson’s correlation analysis. We analyzed the distribution of MSR1 repeat elements with Fisher’s exact test, and we used linear regression analysis to evaluate expression level against age. The level of significance was set to 0.05.

## 3. Results

**Figure 2:**
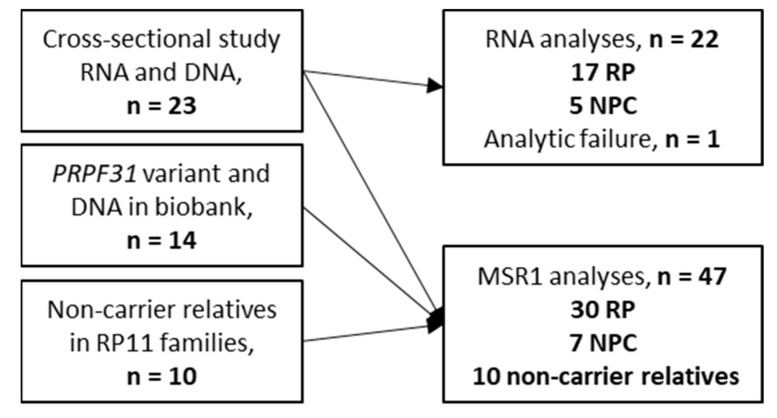
Numbers of analyzed samples and the individuals’ RP status in each step of the study.

We included 37 individuals with a *PRPF31* variant classified as pathogenic or likely pathogenic, 30 had retinitis pigmentosa (RP) and seven were non-penetrant carriers (NPC). In addition, 10 first-degree relatives without the family-specific *PRPF31* variant provided data for the MSR1 segregation analysis, see Figure 2. Twenty-two individuals provided a blood sample that could be used for mRNA expression analyses. The *PRPF31* variants in this study have been previously reported [9], see also Supplement Table S1.

### 3.1. Expression Analyses of PRPF31 and CNOT3

We analyzed 22 samples (5 NPC, 17 RP). The mean ages of the subjects were 44.9 years (SD 18.2) in the RP group, and 47.0 years (SD 23.7) in the NPC group. We found no statistically significant difference in *PRPF31* mRNA expression levels in whole-blood samples between RP patients and non-penetrant carriers. Nor was there a difference in *CNOT3* expression levels, see Table 1. The results were confirmed with the non-parametric Wilcoxon’s rank sum test (*PRPF31* levels, *p* = 0.7; *CNOT3* levels, *p* = 0.5).

**Figure 3:**
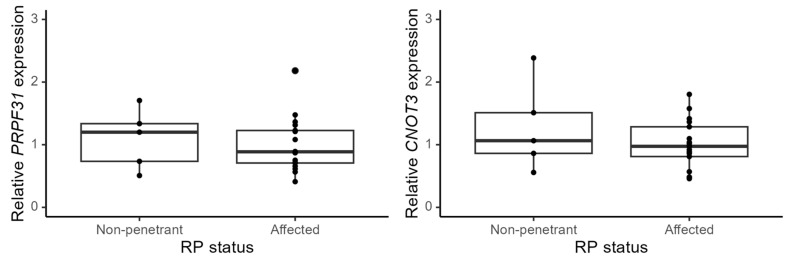
Boxplot of the relative expression in non-penetrant carriers and symptomatic RP individuals (affected). Levels are expressed in fold change with the RP group as reference (mean expression 1.0). *PRPF31* (**left**) and *CNOT3* (**right**). There is no statistical difference in expression levels between the two groups. The endogenous reference gene used was *GAPDH*.

We calculated the fold change using the RP group as a reference and found a mean change of factor 1.1 (*p* = 0.66) in *PRPF31* mRNA expression and a factor 1.3 (*p* = 0.27) change in *CNOT3* mRNA expression. With both genes, the NPC group had a higher mean value than the RP group, but the fold changes were not significant, and ranges were comparable with great overlap, see Figure 3.

The statistical calculations on the mRNA expression analyses were repeated without the two RP individuals carrying the c.666_668del in frame deletion variant p.(Ile223del), because their results might include gene expression from the variant allele, which represent faulty mRNA. However, the results remained unchanged, and we found no statistically significant difference between the RP group and NPC in neither *PRPF31* levels (*p* = 0.69), nor *CNOT3* levels (*p* = 0.36).

**Figure 4:**
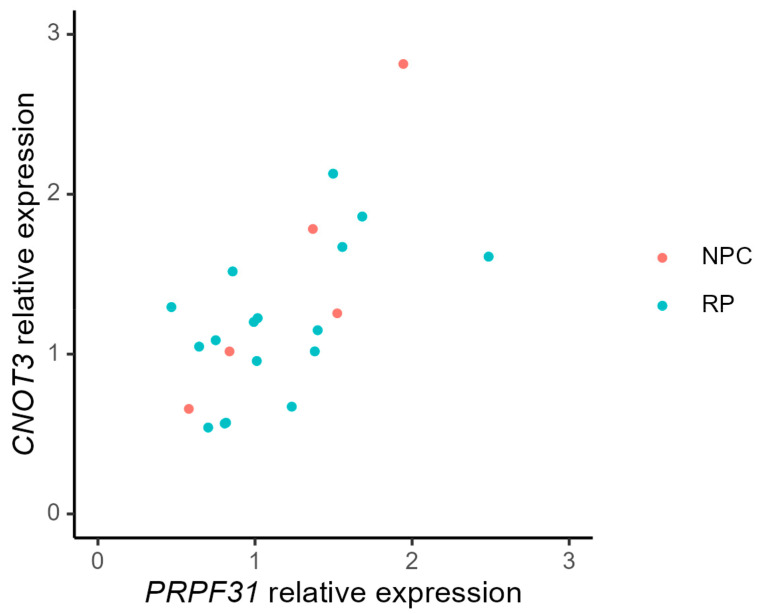
mRNA expression levels in peripheral blood of *CNOT3* and *PRPF31*. The non-penetrant carriers (NPC) are depicted as red dots and individuals with RP as blue dots.

For both the RP individuals and the non-penetrant carriers in our cohort, we found significant positive correlations between *PRPF31* expression levels and *CNOT3* expression levels, see Figure 4. The Pearson correlation coefficient in the RP group was r = 0.55 (*p* = 0.02) and for the NPCs the coefficient was r = 0.9 (*p* = 0.04).

**Figure 5:**
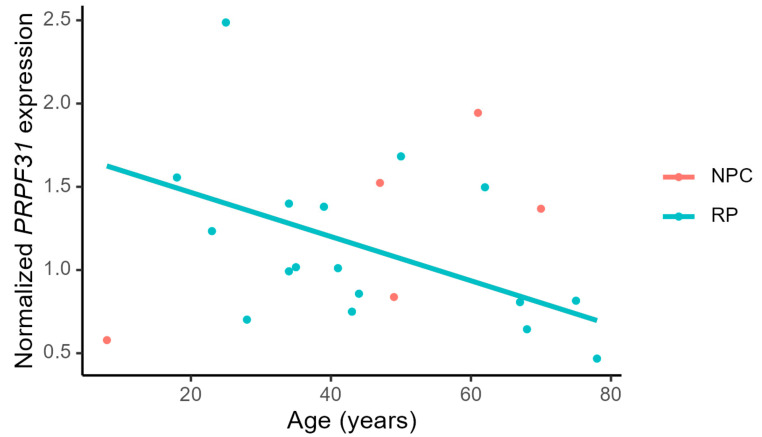
*PRPF31* expression normalized to *GAPDH* plotted against age. There is a borderline significant effect (*p* = 0.04) of age on a decreasing normalized *PRPF31* expression. The linear regression analysis is made on the data from the RP group (blue points), y = −0.013x + 1.73 (R^2^ = 0.25). Levels in non-penetrant carriers (red points) are not significantly changed with age (*p* = 0.16).

The level of *PRPF31* mRNA expression declined with age in affected individuals (*p* = 0.04) but not in non-penetrant carriers, where there was an insignificant increasing tendency (*p* = 0.16), see Figure 5. The level of *CNOT3* mRNA expression did not change with age in any of the groups (data not shown).

### 3.2. MSR1 Analyses

Samples for MSR1 copy numbers were available from 47 subjects (12 families). Ten individuals were non-carriers of the *PRPF31* variant believed to be disease-causing in the family. Of the 37 individuals with pathogenic *PRPF31* variants, 20 had a 3/3 genotype, 16 had a 3/4 genotype, and one individual had a 4/4 genotype. In 3/4 genotype individuals, we used pedigrees to examine segregation and determine whether the 4-copy MSR1 were cis or trans to the *PRPF31* variant, see examples in Supplemental Figure S1. Table 2 summarizes the distribution according to the WT and the mutant allele, respectively. In four related individuals with a 3/4 genotype, all of whom were affected with RP, we were not able to deduct from the pedigree, or from other information available, if the 4-copy allele was the WT or the *PRPF31*-variant allele. These individuals are not included in Table 2 or in the analyses below.

No individuals with symptomatic RP had a 4-copy WT allele. Both 3-copy and 4-copy WT alleles were found in non-penetrant carriers. The risk of symptomatic RP was significantly reduced for those who had a 4-copy WT allele compared to a 3-copy WT allele (Fisher’s exact test, *p* = 0.006). In contrast, MSR1 copy number variation on the *PRPF31* variant allele did not contribute to disease penetrance, and we found no association between RP symptoms and MSR1 CNV on the mutant allele (Fisher’s exact test, *p* = 0.68). Variant alleles containing three or four copies of MSR1 were found in both RP and NPC. Two of the NPC individuals with the 4-copy WT genotype, from whom we had RNA, did not show a higher *PRPF31* expression than other NPCs, and had a normalized *PRPF31* mRNA expression below the average mRNA expression level in the RP group.

## 4. Discussion

Retinitis pigmentosa type 11 is a common cause of autosomal dominant RP that is characterized by the presence of non-penetrant carriers of disease-causing variants in the *PRPF31* gene. The NPC may hold the key to understanding the disease process. Understanding who will develop retinitis pigmentosa and who will remain asymptomatic is of fundamental importance to affected families. Our findings support that having a 4-copy MSR1 on the healthy wild-type allele reduces the risk of developing symptomatic RP11. We found no difference in the *PRPF31* mRNA expression in peripheral blood between non-penetrant carriers and RP11 individuals, suggesting that the *PRPF31* mRNA expression may differ in different tissues, and that whole blood is not the optimal medium for monitoring the *PRPF31* mRNA expression.

We found a clear indication that the 4-copy MSR1 allele is a protective factor against the development of symptomatic RP. This is in agreement with other studies. Four previous studies have analyzed MSR1 CNV in relation to non-penetrance of RP11 [24,26,31,32]. One study found that all 42 RP11 patients carried the MSR1 3/3 genotype and that 8 of the 29 asymptomatic individuals had one 4-copy WT allele [24]. Another study found that all 24 RP individuals had the 3/3 MSR1 genotype, while three out of five non-penetrant carriers had a 3/4 genotype, indicating that a 4-copy allele increased *PRPF31* expression [26]. It was not stated whether the 4-copy allele was on the WT or mutant allele. A different study found that 19/35 symptomatic patients had a 3/4 genotype and that the 4-copy allele was always the mutant allele [31]. The last study reported one NPC with a 3/3 genotype [32]. Altogether, including our study, 51 individuals were found to have a homozygous, hemizygous, or heterozygous 4-copy MSR1 genotype; in 13 of these cases, the 4-copy were definitely on the WT allele, and all these individuals are reportedly non-penetrant. Still, MSR1 copy number is not the sole modifier of *PRPF31* expression or disease penetrance, as not all non-penetrant carriers had a 4-copy MSR1 WT. We discovered no link between the MSR1 distribution and the normalized *PRPF31* mRNA expression values in whole blood. Surprisingly, we observed that the two individuals with the 4-copy WT allele presented with a lower *PRPF31* mRNA expression than the average expression level in the RP group.

The presence or absence of clinical disease in patients with disease-causing variants in *PRPF31* is thought to be related to expression of *PRPF31* from the wild-type allele. We did not find a significant difference in the relative quantification of *PRFP31* mRNA expression levels between RP11 patients and NPC. However, according to the theory of haploinsufficiency, it is precisely a differential expression of *PRPF31* that leads to non-penetrant carriers [20]. A study demonstrated that the relative *PRPF31* mRNA expression in lymphoblastoid cell lines (LCLs) from 200 control individuals varied five-fold [33], indicating that a more highly expressive WT allele might be sufficient to avoid RP. Other studies have used immortalized lymphoblastoid cell lines (LCL) derived from peripheral blood to perform the mRNA expression analyses in carriers of *PRPF31* variants, and demonstrated a significant difference in *PRPF31* expression; with non-penetrant carriers having significantly higher mRNA expression levels than RP individuals [7,25,33,34,35,36]. This is clearly exemplified by one study (n = 7 RP + 3 NPC) which reported a 29–42% increase in *PRPF31* expression in NPCs compared to RP individuals [35], and another study (n = 8 RP + 7 NPC) demonstrated a significant difference (*p* < 0.001) in mRNA copy number between the two groups [34]. Smaller studies (n = 5 RP + 6 NPC and n = 2 RP + 1 NPC), also using LCLs, found only a borderline significant difference [37] or no significant differences in *PRPF31* mRNA expression [32], respectively.

We chose an assay based on whole peripheral blood to test if a simple blood test could be a useful indicator of disease, and to avoid bias from selection of specific cell lines when transforming the cells to lymphoblastoid cell lines [38]. A blood sample is easily applicable, quick to analyze, and could hypothetically be supportive when counseling patients and families with RP. However, *PRPF31* mRNA expression levels differ between different cell types (retinal organoids, retinal pigment epithelium, retinal tissue, and fibroblasts) [26]. The *PRPF31* expression level in fibroblasts is reportedly lower in RP11 subjects than in WT controls, but not lower than the levels in NPC [26]. Two studies have investigated *PRPF31* expression from total RNA in blood, similar to our approach, and found that the levels in NPC were intermediate between RP individuals and the control group (n = 7 RP, 3 NPC, 8 controls) [39], and that *PRPF31* expression levels were reduced in RP compared to controls (n = 3 RP, 9 controls, no NPC) [31]. 

Previous studies found that *CNOT3* expression had an inverse correlation with *PRPF31*, suggesting that *CNOT3* might have an inhibiting effect on *PRPF31* expression [25,40]. Particularly, the *CNOT3* rs4806718 polymorphism was suspected to correlate with non-penetrance, demonstrated in a study from 2012, which found a significant correlation (*p* = 0.04) [25]. Moreover, one study found, by linkage analysis, that inheritance of two “high expressive” *CNOT3* alleles was associated with symptomatic RP [40]. Two other studies have investigated *CNOT3* expression and found no correlation between non-penetrance and the rs4806718 polymorphism [26,32]. In our study we did not investigate specifically for the *CNOT3* rs4806718 polymorphism, but we found *CNOT3* mRNA expression to have a positive correlation with *PRPF31* expression, and that *CNOT3* expression levels were not correlated with non-penetrance. This was in line with the recent studies [26,32].

The contradictory results suggest that the disease mechanism for RP11 is not fully mapped and that there is a multifactorial basis for the phenotype, although WT gene expression is considered to be the primary cause [41]. Other genetic modifiers, in addition to those discussed in this paper, may affect the WT gene expression; for instance, not all NPCs carried an MSR1 4-copy WT allele. Moreover, dominant-negative effects of some genetic variants have been suggested to affect disease status [42,43]. In addition, the impact of age on *PRPF31* gene expression and its contribution to the phenotype is also of interest. One study showed that expression levels of *PRPF31* declined with age in retinal tissue [26], and our study highlighted a declining expression level in whole blood in RP11 subjects. In theory, this could imply that mild or subclinical disease might develop with age in some apparently NPC individuals. Our age analysis was only borderline significant, and if we omitted just one data point, the highest expression value, we would have lost significance and the implication of age is uncertain. Further research is needed to fully understand the complex interplay between various factors and their contribution to WT gene expression and to the phenotype of the disease.

The method used, RNA isolated from whole blood, has only been applied in very small RP11-cohorts and it is uncertain how blood expression levels reflect the eye tissue expression levels, which was one of the study’s limitations. Recruiting participants with a rare disease can be a challenge. Our study has a comparable or higher number of participants than other studies on *PRPF31* expression analyses, but it is still a small cohort, and a larger sample size would have been beneficial. Furthermore, it would have been interesting to include non-carrier relatives or control subjects in the expression analysis, as well as to compare mRNA expression from other mediums across the same subjects. It was a limitation that we could only use subjective symptoms to identify NPCs, since we did not rule out or comment on potentially mild structural retinal changes. The primers used in this study were not specifically designed to circumvent disease-causing variants, which, in theory, means that our expression levels may have included defunct mRNA. We expect this risk to be low given that the vast majority of *PRPF31* variants in our cohort result in null alleles, and also after the in frame deletion variant p.(Ile223del), which is not expected to lead to NMD, was omitted from the analysis, the results remained unchanged.

In conclusion, expression levels of *PRPF31* mRNA in whole blood does not reflect disease penetrance in our cohort, and suggests, unfortunately, that a blood sample alone cannot be used to predict non-penetrance in RP11. Nevertheless, this study supports that a 4-copy MSR1 CNV cis with the WT allele is associated with non-penetrance of RP11 disease. To date symptomatic disease has not been described in individuals with a 4-copy MSR1 CNV on the wild-type allele. This suggests that the WT 4-copy MSR1 variant is a safe indicator of non-penetrance and may prove a useful tool in family counseling, which is particularly useful for family members in pre-symptomatic testing.

## Figures and Tables

**Table 1:** Relative mean expression values, normalized to *GAPDH*, and unpaired two-tailed *t*-test significance between the groups.

	RP Group	Non-Penetrant Carriers	Fold Change	*p*-Value
*PRPF31*, relative normalized mean ± SD	1.14 ± 0.50	1.25 ± 0.55	1.1	0.66
*CNOT3*, relative normalized mean ± SD	1.18 ± 0.46	1.51 ± 0.84	1.3	0.27

**Table 2:** MSR1 CNV on the wild-type allele and the mutant allele in individuals with a *PRPF31*-variant.

MSR1 on Wild-Type Allele	RP	NPC
4-copy on WT, n	0	3
3-copy on WT, n	26	4
**MSR1 on *PRPF31* Variant Allele**	RP	NPC
4-copy on mutant, n	11	2
3-copy on mutant, n	15	5

## Data Availability

The data presented in this study are available on reasonable request from the corresponding author.

## Supplementary Materials

The following supporting information can be downloaded at https://www.mdpi.com/article/10.3390/genes14020435/s1, Figure S1: Pedigree examples; Table S1: *PRPF31* variants.

## References

[1] Vithana E.N., Abu-Safieh L., Allen M.J., Carey A., Papaioannou M., Chakarova C., Al-Maghtheh M., Ebenezer N.D., Willis C., Moore A.T. (2001). A Human Homolog of Yeast Pre-MRNA Splicing Gene, PRP31, Underlies Autosomal Dominant Retinitis Pigmentosa on Chromosome 19q13.4 (RP11). Mol. Cell.

[2] Verbakel S.K., van Huet R.A.C., Boon C.J.F., den Hollander A.I., Collin R.W.J., Klaver C.C.W., Hoyng C.B., Roepman R., Klevering B.J. (2018). Non-Syndromic Retinitis Pigmentosa. Prog. Retin. Eye Res..

[3] Ferrari S., Di Iorio E., Barbaro V., Ponzin D., Sorrentino F.S., Parmeggiani F. (2011). Retinitis Pigmentosa: Genes and Disease Mechanisms. Curr. Genom..

[4] Haim M. (2002). The Epidemiology of Retinitis Pigmentosa in Denmark. Acta Ophthalmol. Scand. Suppl..

[5] Hartong D.T., Berson E.L., Dryja T.P. (2006). Retinitis Pigmentosa. Lancet.

[6] Dias M.F., Joo K., Kemp J.A., Fialho S.L., da Silva Cunha A., Woo S.J., Kwon Y.J. (2018). Molecular Genetics and Emerging Therapies for Retinitis Pigmentosa: Basic Research and Clinical Perspectives. Prog. Retin. Eye Res..

[7] Ruberto F.P., Balzano S., Namburi P., Kimchi A., Pescini-Gobert R., Obolensky A., Banin E., Ben-Yosef T., Sharon D., Rivolta C. (2021). Heterozygous Deletions of Noncoding Parts of the PRPF31 Gene Cause Retinitis Pigmentosa via Reduced Gene Expression. Mol. Vis..

[8] Abu-Safieh L., Vithana E.N., Mantel I., Holder G.E., Pelosini L., Bird A.C., Bhattacharya S.S. (2006). A Large Deletion in the AdRP Gene PRPF31: Evidence That Haploinsufficiency Is the Cause of Disease. Mol. Vis..

[9] Lisbjerg K., Bertelsen M., Lyng Forman J., Grønskov K., Prener Holtan J., Kessel L. (2022). Disease Progression of Retinitis Pigmentosa Caused by PRPF31 Variants in a Nordic Population: A Retrospective Study with up to 36 Years Follow-Up. Ophthalmic Genet..

[10] Kiser K., Webb-Jones K.D., Bowne S.J., Sullivan L.S., Daiger S.P., Birch D.G. (2019). Time Course of Disease Progression of PRPF31-Mediated Retinitis Pigmentosa. Am. J. Ophthalmol..

[11] Moore A.T., Fitzke F., Jay M., Arden G.B., Inglehearn C.F., Keen T.J., Bhattacharya S.S., Bird A.C. (1993). Autosomal Dominant Retinitis Pigmentosa with Apparent Incomplete Penetrance: A Clinical, Electrophysiological, Psychophysical, and Molecular Genetic Study. Br. J. Ophthalmol..

[12] Russell S., Bennett J., Wellman J.A., Chung D.C., Yu Z.F., Tillman A., Wittes J., Pappas J., Elci O., McCague S. (2017). Efficacy and Safety of Voretigene Neparvovec (AAV2-HRPE65v2) in Patients with RPE65-Mediated Inherited Retinal Dystrophy: A Randomised, Controlled, Open-Label, Phase 3 Trial. Lancet.

[13] Thompson D.A., Iannaccone A., Ali R.R., Arshavsky V.Y., Audo I., Bainbridge J.W.B., Besirli C.G., Birch D.G., Branham K.E., Cideciyan A.V. (2020). Advancing Clinical Trials for Inherited Retinal Diseases: Recommendations from the Second Monaciano Symposium. Transl. Vis. Sci. Technol..

[14] Rose A.M., Luo R., Radia U.K., Bhattacharya S.S. (2017). Gene of the Month: PRPF31. J. Clin. Pathol..

[15] Hoskins A.A., Friedman L.J., Gallagher S.S., Crawford D.J., Anderson E.G., Wombacher R., Ramirez N., Cornish V.W., Gelles J., Moore M.J. (2011). Ordered and Dynamic Assembly of Single Spliceosomes. Science.

[16] Tanackovic G., Ransijn A., Thibault P., Elela S.A., Klinck R., Berson E.L., Chabot B., Rivolta C. (2011). PRPF Mutations Are Associated with Generalized Defects in Spliceosome Formation and Pre-MRNA Splicing in Patients with Retinitis Pigmentosa. Hum. Mol. Genet..

[17] Tanackovic G., Rivolta C. (2009). PRPF31 Alternative Splicing and Expression in Human Retina. Ophthalmic Genet..

[18] Linder B., Dill H., Hirmer A., Brocher J., Lee G.P., Mathavan S., Bolz H.J., Winkler C., Laggerbauer B., Fischer U. (2011). Systemic Splicing Factor Deficiency Causes Tissue-Specific Defects: A Zebrafish Model for Retinitis Pigmentosa. Hum. Mol. Genet..

[19] Yusuf I.H., Birtel J., Shanks M.E., Clouston P., Downes S.M., Charbel Issa P., Maclaren R.E. (2019). Clinical Characterization of Retinitis Pigmentosa Associated with Variants in SNRNP200. JAMA Ophthalmol..

[20] Rose A.M., Bhattacharya S.S. (2016). Variant Haploinsufficiency and Phenotypic Non-Penetrance in PRPF31-Associated Retinitis Pigmentosa. Clin. Genet..

[21] Wheway G., Douglas A., Baralle D., Guillot E. (2020). Mutation Spectrum of PRPF31, Genotype-Phenotype Correlation in Retinitis Pigmentosa, and Opportunities for Therapy. Exp. Eye Res..

[22] Roshandel D., Thompson J.A., Jeffery R.C.H., Zhang D., Lamey T.M., McLaren T.L., De Roach J.N., McLenachan S., Mackey D.A., Chen F.K. (2021). Clinical Evidence for the Importance of the Wild-Type Prpf31 Allele in the Phenotypic Expression of Rp11. Genes.

[23] McGee T.L., Devoto M., Ott J., Berson E.L., Dryja T.P. (1997). Evidence That the Penetrance of Mutations at the RP11 Locus Causing Dominant Retinitis Pigmentosa Is Influenced by a Gene Linked to the Homologous RP11 Allele. Am. J. Hum. Genet..

[24] Rose A.M., Shah A.Z., Venturini G., Krishna A., Chakravarti A., Rivolta C., Bhattacharya S.S. (2016). Transcriptional Regulation of PRPF31 Gene Expression by MSR1 Repeat Elements Causes Incomplete Penetrance in Retinitis Pigmentosa. Sci. Rep..

[25] Venturini G., Rose A.M., Shah A.Z., Bhattacharya S.S., Rivolta C. (2012). CNOT3 Is a Modifier of PRPF31 Mutations in Retinitis Pigmentosa with Incomplete Penetrance. PLoS Genet..

[26] McLenachan S., Zhang D., Grainok J., Zhang X., Huang Z., Chen S.C., Zaw K., Lima A., Jennings L., Roshandel D. (2021). Determinants of Disease Penetrance in Prpf31-Associated Retinopathy. Genes.

[27] Jespersgaard C., Fang M., Bertelsen M., Dang X., Jensen H., Chen Y., Bech N., Dai L., Rosenberg T., Zhang J. (2019). Molecular Genetic Analysis Using Targeted NGS Analysis of 677 Individuals with Retinal Dystrophy. Sci. Rep..

[28] Richards S., Aziz N., Bale S., Bick D., Das S., Gastier-Foster J., Grody W.W., Hegde M., Lyon E., Spector E. (2015). Standards and Guidelines for the Interpretation of Sequence Variants: A Joint Consensus Recommendation of the American College of Medical Genetics and Genomics and the Association for Molecular Pathology. Genet. Med..

[29] R Core Team (2021). R: A Language and Environment for Statistical Computing.

[30] De Winter J.C.F. (2013). Using the Student’s t-Test with Extremely Small Sample Sizes. Pract. Assessment Res. Eval..

[31] Martin-Merida I., Sanchez-Alcudia R., Jose P.F.S., Blanco-Kelly F., Perez-Carro R., Da Silva L.R.J., Almoguera B., Garcia-Sandoval B., Lopez-Molina M.I., Avila-Fernandez A. (2017). Analysis of the PRPF31 Gene in Spanish Autosomal Dominant Retinitis Pigmentosa Patients: A Novel Genomic Rearrangement. Investig. Ophthalmol. Vis. Sci..

[32] Ali-nasser T., Zayit-soudry S., Banin E., Sharon D., Ben-yosef T., Ali T. (2022). Autosomal Dominant Retinitis Pigmentosa with Incomplete Penetrance Due to an Intronic Mutation of the PRPF31 Gene. Mol. Vis..

[33] Rio Frio T., Civic N., Ransijn A., Beckmann J.S., Rivolta C. (2008). Two Trans-Acting EQTLs Modulate the Penetrance of PRPF31 Mutations. Hum. Mol. Genet..

[34] Vithana E.N., Abu-Safieh L., Pelosini L., Winchester E., Hornan D., Bird A.C., Hunt D.M., Bustin S.A., Bhattacharya S.S. (2003). Expression of PRPF31 MRNA in Patients with Autosomal Dominant Retinitis Pigmentosa: A Molecular Clue for Incomplete Penetrance?. Investig. Ophthalmol. Vis. Sci..

[35] Rivolta C., McGee T.L., Frio T.R., Jensen R.V., Berson E.L., Dryja T.P. (2006). Variation in Retinitis Pigmentosa-11 (PRPF31 or RP11) Gene Expression between Symptomatic and Asymptomatic Patients with Dominant RP11 Mutations. Hum. Mutat..

[36] Frio T.R., Wade N.M., Ransijn A., Berson E.L., Beckmann J.S., Rivolta C. (2008). Premature Termination Codons in PRPF31 Cause Retinitis Pigmentosa via Haploinsufficiency Due to Nonsense-Mediated MRNA Decay. J. Clin. Investig..

[37] Villanueva A., Willer J.R., Bryois J., Dermitzakis E.T., Katsanis N., Davis E.E. (2014). Whole Exome Sequencing of a Dominant Retinitis Pigmentosa Family Identifies a Novel Deletion in PRPF31. Investig. Ophthalmol. Vis. Sci..

[38] Lenartowicz M., Moos T., Ogórek M., Jensen T.G., Møller L.B. (2016). Metal-Dependent Regulation of ATP7A and ATP7B in Fibroblast Cultures. Front. Mol. Neurosci..

[39] Liu J.Y., Dai X., Sheng J., Cui X., Wang X., Jiang X., Tu X., Tang Z., Bai Y., Liu M. (2008). Identification and Functional Characterization of a Novel Splicing Mutation in RP Gene PRPF31. Biochem. Biophys. Res. Commun..

[40] Rose A.M., Shah A.Z., Venturini G., Rivolta C., Rose G.E., Bhattacharya S.S. (2014). Dominant PRPF31 Mutations Are Hypostatic to a Recessive CNOT3 Polymorphism in Retinitis Pigmentosa: A Novel Phenomenon of “Linked Trans -Acting Epistasis”. Ann. Hum. Genet..

[41] Yang C., Georgiou M., Atkinson R., Collin J., Al-Aama J., Nagaraja-Grellscheid S., Johnson C., Ali R., Armstrong L., Mozaffari-Jovin S. (2021). Pre-MRNA Processing Factors and Retinitis Pigmentosa: RNA Splicing and Beyond. Front. Cell Dev. Biol..

[42] Yin J., Brocher J., Fischer U., Winkler C. (2011). Mutant Prpf31 Causes Pre-MRNA Splicing Defects and Rod Photoreceptor Cell Degeneration in a Zebrafish Model for Retinitis Pigmentosa. Mol. Neurodegener..

[43] Valdés-Sánchez L., Calado S.M., de la Cerda B., Aramburu A., Belén García-Delgado A., Massalini S., Montero-Sánchez A., Bhatia V., Rodríguez-Bocanegra E., Diez-Lloret A. (2020). Retinal Pigment Epithelium Degeneration Caused by Aggregation of PRPF31 and the Role of HSP70 Family of Proteins. Mol. Med..

