# Supplementary material for: Genetic Modifiers of Non-Penetrance and RNA Expression Levels in PRPF31-Associated Retinitis Pigmentosa in a Danish Cohort
Source: Genes (Basel). 2023 Feb 8;14(2):435. doi: 10.3390/genes14020435 (PMC9956082; doi:10.3390/genes14020435)
Supplement: Supplementary file 1 [file genes-14-00435-s001.zip › genes-2160513-supplementary.pdf]

Supplementary material

Table S1. *PRPF31*-variants identified in this study.18 individuals with RP and 5 non-penetrant carriers were included for mRNA analyses. 37 individuals + 10 non-carrier relatives were included for MSR1 analyses. The genetic variants have previously been published [1].

***PRPF31* variant**			**n, mRNA expression RP (NPC)**	**n, MSR1**
**Nucleotide change**	**Amino acid change**	**Classification (ACMG)**		
c.421-2A>G	p.?	Likely pathogenic (PVS1, PM2_sup)	3	7
c.666_668del	p.(Ile223del)	VUS (PM2_sup, PM4)	2	3
c.828_829delCA	p.(His276Glnfs*2)	Pathogenic (PVS1, PS4_sup, PM2_sup)	1	1
c.855+1G>T	p.?	Likely pathogenic (PVS1, PM2_sup)	1 (1)	3
c.1234delG	p.(Val412*)	Likely pathogenic (PVS1, PM2_sup)	3 (2)	5
c.(-9+1_1-1)_(420+1_421-1)dup	Duplication exons 2-5	Likely pathogenic (PVS1_str, PS4_sup, PP1)	3	4
c.(-9+1_1-1)_(238+1_239-1)del	Deletion exons 2-3	Pathogenic (PVS1, PM2_sup, PS4)	1	5
g.(54619177_54621659)_(54634972_?)del	Deletion exons 2-14	Pathogenic (PVS1, PM2_sup, PS4)	4 (2)	6
c.1190dup	p.(His398Profs*77)	Pathogenic (PVS1, PS4_sup, PM2_sup)	-	1
c.961A>T	p.(Lys321*)	Likely pathogenic (PVS1, PM2_sup)	-	1
c.997del	p.(Glu333Serfs*5)	Pathogenic (PVS1, PS4_sup, PM2_sup)	-	1

PRPF31: NM_015629.4, VUS: variant of unknown significance, sup: supportive, str: strong. n refers to the number of subjects included in the respective analysis.

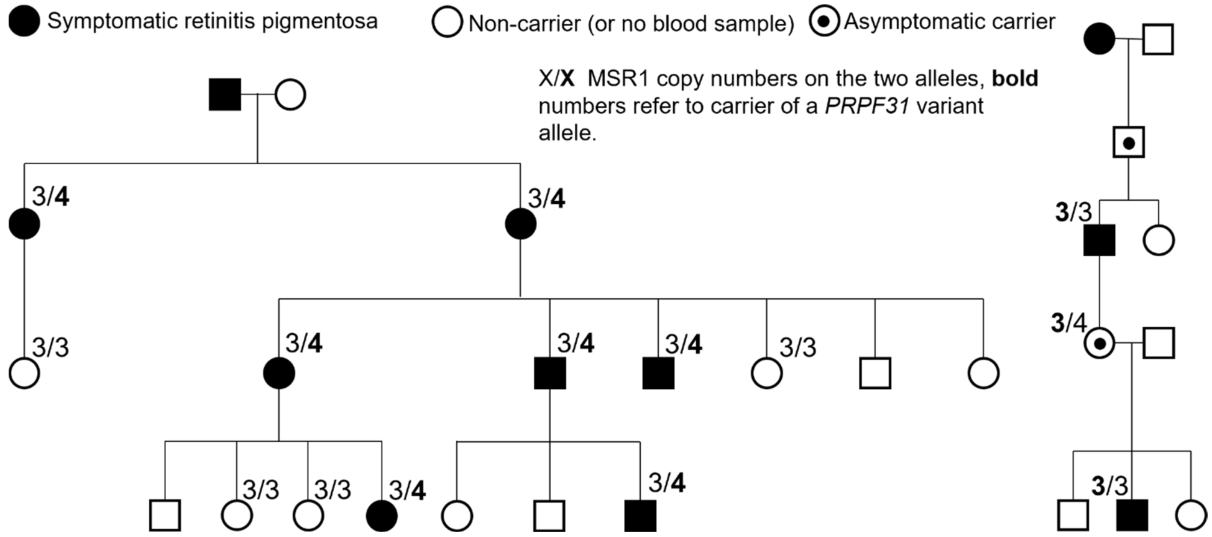

Figure S1. Pedigrees showing examples from two of the RP11 families. In those individuals who had a sample stored in the DNA-bank the two allele's MSR1 copy number were analyzed and highlighted with numbers in the figure. In family 1 (left), the *PRPF31*-variant was on a 4-copy allele, as non-carriers had 3/3 alleles. In family 2 (right), the *PRPF31*-variant was on the 3-copy allele demonstrated by the segregation with a non-penetrant carrier, carrying a 4-copy WT allele, between two affected individuals with a 3/3 genotype.

1. Lisbjerg, K.; Bertelsen, M.; Lyng Forman, J.; Grønskov, K.; Prener Holtan, J.; Kessel, L. Disease Progression of Retinitis Pigmentosa Caused by PRPF31 Variants in a Nordic Population: A Retrospective Study with up to 36 Years Follow-Up. *Ophthalmic Genet.* **2022**, 1–8. https://doi.org/10.1080/13816810.2022.2123006.
